# Predicting vertical ground reaction forces from 3D accelerometry using reservoir computers leads to accurate gait event detection

**DOI:** 10.3389/fspor.2022.1037438

**Published:** 2022-10-26

**Authors:** Margit M. Bach, Nadia Dominici, Andreas Daffertshofer

**Affiliations:** Department of Human Movement Sciences, Faculty of Behavioural and Movement Sciences, Amsterdam Movement Sciences and Institute of Brain and Behaviour Amsterdam, Vrije Universiteit Amsterdam, Amsterdam, Netherlands

**Keywords:** accelerometer, gait detection, ground reaction forces, locomotion, reservoir computing

## Abstract

Accelerometers are low-cost measurement devices that can readily be used outside the lab. However, determining isolated gait events from accelerometer signals, especially foot-off events during running, is an open problem. We outline a two-step approach where machine learning serves to predict vertical ground reaction forces from accelerometer signals, followed by force-based event detection. We collected shank accelerometer signals and ground reaction forces from 21 adults during comfortable walking and running on an instrumented treadmill. We trained one common reservoir computer using segmented data using both walking and running data. Despite being trained on just a small number of strides, this reservoir computer predicted vertical ground reaction forces in continuous gait with high quality. The subsequent foot contact and foot off event detection proved highly accurate when compared to the gold standard based on co-registered ground reaction forces. Our proof-of-concept illustrates the capacity of combining accelerometry with machine learning for detecting isolated gait events irrespective of mode of locomotion.

## Introduction

Estimating the presence of a step using mobile devices can be realized with fair accuracy and relative ease (1–5). Yet, many details of the stepping cycle remain opaque such as foot contact and foot off moments, but also more detailed gait characteristics, such as loading responses in (ambulant) clinical contexts. In most of the current literature on wearables, event estimations are rule-based and often require searching for an area of interest (6, 7). This is true for data from inertial measurement units but also for data derived from only accelerometers. Algorithms are optimized for either walking (8–15) or running (16–23) and vary depending on sensor location and type, and on speed. As it stands, they may not generalize to other settings.

Machine learning approaches may provide welcome alternatives. They have been employed to predict stepping moments and gait phases by extracting different features recorded from inertial measurement units (24–37), 3D marker data (38–41), electromyography (42), pressure sensors (43), and textile sensors (44). Across the board, though, these approaches required a priori feature extractions and are, hence, potentially jeopardized by selection bias.

Stepping instants can readily be identified using (the shape of) ground reaction forces (GRFs), typically obtained from force plate signals (45, 46). With these, one can specify single/double support and flight phases and, correspondingly, the mode of locomotion, i.e., walking or running. As such it seems obvious to first seek to estimate the GRF's shape from wearable sensors and to subsequently use these predicted waveforms to determine gait events. Also here, machine learning has been successful. GRFs during the stance phase, for instance, has been estimated using only acceleration (47–50), a combination of acceleration and angular velocity (51–54), or marker-based kinematics (55, 56). The GRFs during double stance phase could be estimated via marker-based kinematics (57, 58) and the GRFs during the full gait cycle using accelerometers placed on the torso (59). Yet, these approaches often appeared tailored to the data under study rendering their generalizability questionable, but more importantly, in almost all cases, they only managed to predict GRFs for the stance phase, whereas the (duration of the) swing phase is of great importance when investigating running.

A recent review revealed that the shape of the GRF can most accurately be estimated from accelerometry (60) and another found neural networks as a promising tool to do so (61). This triggered the idea of estimating vertical GRF waveforms from acceleration signals of the lower extremities via reservoir computers, more specifically via echo state networks (ESNs) (62–64). ESNs are “minimal” forms of recurrent neural networks. Thanks to the reservoir's “complex” structure, they may come with great computational capacities (65, 66). In the absence of feedback, one can train them with a very simple and robust rule: optimizing output weights by mere linear regression. This is particularly appealing when considering that typical data sets on gait are fairly limited in size and that any implementation of machine learning in wearable devices should come with low computational costs.

In the following, we conceptually prove that a single reservoir computer can accurately predict vertical GRF waveforms from shank accelerometer signals, which allows for detecting gait events during walking and running with particularly high precision.

## Methods

### Participants

We included data of 21 healthy young adults (13 male/8 female) in the analysis with a mean ± standard deviation age, height, and weight of 20.8 ± 1.0 years, 181.7 ± 10.3 cm, 71.1 ± 9.8 kg, respectively. The recorded speeds were 1.24 ± 0.12 m/s for walking and 2.20 ± 0.14 m/s for running. The participants provided written informed consent in compliance with the Declaration of Helsinki. The experimental design was approved by The Scientific and Ethical Review Board of the Faculty of Behavioural and Movement Sciences, Vrije Universiteit Amsterdam, Netherlands (File number: VCWE-2022-008R1).

### Experimental protocol

Participants walked and ran at their preferred speeds on an instrumented dual-belt treadmill (Motek Medical BV, Culemborg, Netherlands) in tied-belt mode wearing their own shoes. The preferred walking and running speeds were determined for each participant followed by a familiarization. The preferred speeds were determined by starting at either 2 km/h or 6.5 km/h for walking and running, respectively, and slowly increasing the speed by 0.1 km/h until the participant felt it was comfortable (67). Subsequently, the same process was repeated at a speed 1.5 km/h above this by now slowly decreasing the speed by 0.1 km/h until a new or the same preferred speed was reached. If the two speeds differed more than 0.4 km/h from each other, a third iteration was done, and irrespective of two or three iterations, the mean of the determined preferred speeds was used. The participants were instructed to step with each foot on a separate belt to be able to record the time series of the ground reaction forces from one leg. For each participant a walking and a running trial were recorded of each 5 mins in length. Only consecutive strides absent of artifacts (stepping on the wrong belt) were retained leaving an average of 72 strides per trial for analysis (range: 49–116 strides).

**Figure 1:**
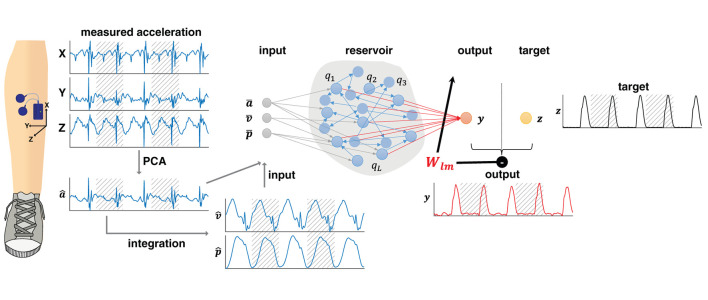
A reservoir computer was implemented to predict the vertical ground reaction forces. Tri-axial accelerometer data were recorded from the shank. The accelerometer data were re-oriented using a principal component analysis (PCA). The first prinicipal component (â) was integrated once to obtain the velocity ($\hat{v}$) and position ($\hat{p}$) data. The input **x** consisting of the normalized accelerometer ($\bar{a})$, velocity ($\bar{v})$, and position ($\bar{p})$ data were subsequently mapped onto the sparsely, randomly connected reservoir **q**. This reservoir generated the output **y**, the predicted normalized vertical ground reaction forces (in red), via output weights **W**. When training, the output was compared to the target **z**, i.e., the measured vertical ground reaction forces (in black). Minimizing the difference between generated output and target served to adjust the weights (denoted here as **W**_**lm**_). For training, data were segmented into strides, here represented by hatched and unhatched areas. Testing was conducted on continuous data. Data from walking and running were pooled.

Tri-axial accelerometers, built into the probe of the wireless bipolar surface electromyography system (Mini wave plus, Zerowire; Cometa, Bareggio, Italy), were mounted on the right and left tibia, respectively (see Figure 1). The accelerometers were not placed in the same exact position due to inexperienced researchers. Accelerometer data were sampled at 2,000/14 Hz (=142.86 Hz) which in the following will be referred to as ~143 Hz, with a sensitivity of ± 16 g, which was sufficient to avoid clipping. The vertical GRFs were sampled at 1 kHz and re-sampled to ~143 Hz.

A single reservoir computer was trained to predict ipsilateral continuous vertical ground reaction forces based on the shank accelerometer data recorded during walking and running. Figure 1 contains a schematic of the pre-processing steps and the implemented machine learning approach. Further details are presented in the following.

### Data processing

Accelerometer signals *a* were first “standardized” to their principal axes using principal component analysis (PCA) (68, 69):


$$\begin{array}{l} a=\left(a_{x},a_{y},a_{z}\right)\to^{PCA}â \end{array}$$

with â along the direction of maximum variance and being the only principal component that was retained. â was integrated twice over time (after a bi-directional high pass Butterworth filter with cut-off at 1 Hz, 2nd order) to generate likewise standardized velocities $\hat{v}$ and positional data $\hat{p}$:


$$\begin{array}{l} \hat{v}=\int_{0}^{t}â\cdot dt\;and\;\hat{p}=\int_{0}^{t}\hat{v}\cdot dt \end{array}$$

Per subset (trial) these signals were normalized (70) by means of


$$\begin{array}{ll} â\to \bar{a} & =\frac{â}{\text{range}\left(â\right)}\text{ and }\\ \;\hat{v}\to \bar{v} & =\frac{\hat{v}}{\text{range}\left(\hat{v}\right)}\text{ and }\hat{p}\to \bar{p}=\frac{\hat{p}}{\text{range}\left(\hat{p}\right)} \end{array}$$

before combining them as three-dimensional input data


$$\begin{array}{l} x=\left(\bar{a},\bar{v},\bar{p}\right)\in {\mathbb{R}}^{3\times T} \end{array}$$

with *T* indicating the number of samples in time. Vertical ground reaction forces *F*_*z*_ were normalized. With this, the target signal for our machine learner (see below) could be defined as z-score (70).


$$\begin{array}{l} z={\bar{F}}_{z}=\frac{\left(F_{z}-\mu \left(F_{z}\right)\right)}{\sigma \left(F_{z}\right)}\in {\mathbb{R}}^{1\times T} \end{array}$$

with μ and σ denoting the mean and standard deviation over time per trial.

Stepping moments (foot contact and foot off events) were identified based on the measured *F*_*z*_ through mere thresholds: first, the *F*_*z*_ was scaled to a range [0 1], then weakly filtered with a polynomial Savitzky-Golay filter (1st order, ± 30 ms = in total 9 samples) (71). The foot contact was defined as the last point below a threshold (12.5% of the maximum of the data) nearest the ascend of the *F*_*z*_ of the stance phase; similarly, the foot off was defined as the first point crossing the same threshold nearest the descending *F*_*z*_ (46, 72).

Data were split according to the defined foot off events for further analysis. We considered 36 samples in time on either side of the foot off as transients when correcting for learning errors in the beginning or end of the data. These transients also served to ensure that data were independent of the true events as 36 samples represent different percentage of the stride for walking and running, respectively.

### Reservoir computer

We adopted the leaky ESN implementation by Jaeger and Haas (62) [see also (73, 74)]. In brief, we built a reservoir of *N* nodes $q=\left(q_{1},q_{2},\ldots ,q_{N}\right)\in {\mathbb{R}}^{N\times T}$ that received an input $x=\left(x_{1},x_{2},\ldots ,x_{K}\right)\in {\mathbb{R}}^{K\times T}$ and generated output $y=\left(y_{1},y_{2},\ldots ,y_{M}\right)\in {\mathbb{R}}^{M\times T}$. During the supervised training mode output was compared with target $z=\left(z_{1},z_{2},\ldots ,z_{M}\right)\in {\mathbb{R}}^{M\times T}$ by means of the L_2_-norm (75) (cf. Figure 1).

The reservoir dynamics can be written as


$$\begin{array}{l} dq=\tau^{-1}\left[-\gamma q+\text{tanh}\left(Cq+Fx\right)\right]dt+d\varepsilon \end{array}$$

where *C*∈ℝ^*N*×*N*^ denotes the connectivity of the reservoir. Here, *C* was set as a sparse, random matrix specified by a sparseness parameter; its weights were normalized for a given spectral radius (the relative magnitude of the leading eigenvalue of *C*). *F*∈ℝ^*N*×*K*^ was set to be a dense matrix allowing for an optional scaling of the input values when mapping them onto the reservoir. The quantity ε stands for uniformly distributed, uncorrelated noise, i.e., ε∈εU(−1,1), with ε being reasonably small. The output is given by


$$\begin{array}{l} y=Wq \end{array}$$

with *W*∈ℝ^*M*×*N*^, which is the matrix of the to-be-learned output weights.

Learning was realized by ridge regression, i.e.,


$$\begin{array}{l} ||z-q||=||z-Wq|{|}_{2}\to \min\Rightarrow W=Q^{-1}z \end{array}$$

where *Q* = [*q*_1_, …, *q*_*T*_] and (·)^−1^ denotes the pseudo-inverse. In the case of multiple time series, i.e., *S* steps (see below), we defined $Q=\left[q_{1}^{\left(1\right)},\ldots ,q_{T_{1}}^{\left(1\right)};q_{1}^{\left(2\right)},\ldots ,q_{T_{2}}^{\left(2\right)};\ldots ;q_{1}^{\left(S\right)},\ldots ,q_{T_{S}}^{\left(S\right)}\right]$ and accordingly we used *z* = [*z*^(1)^; …;*z*^(*S*)^ ].

The network parameters were set as follows: *N* = 1, 000, spectral radius = 0.5, *F* = [0.1;0.5], τ = 1, γ = 0.5 and ε = 10^−4^. The noise was primarily added to minimize the risk of overfitting and we put ε = 0 after learning.

#### Stepping moments from the predicted vertical ground reaction forces

Stepping moment identification of the predicted vertical ground reaction force waveforms was implemented in the same way as for the measured vertical ground reaction forces (see above).

**Figure 2:**
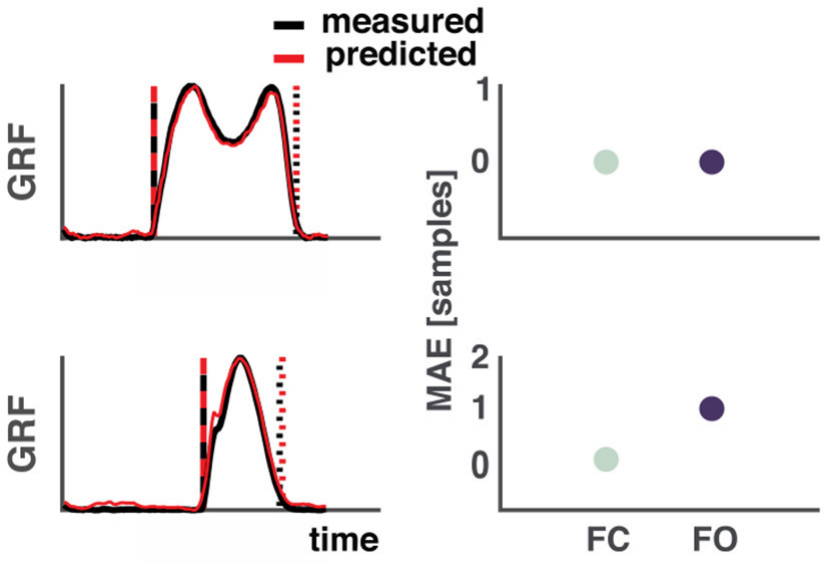
Example of the estimation of foot contact and foot off events from measured and predicted ground reaction forces. **Top**: Walking, **bottom**: Running. **Left side**: Measured vertical GRF waveforms are depicted in black and the predicted ones in red. The vertical dashed lines represent foot contact (black: measured, red: predicted) and the dotted lines represent foot off (black: measured, red: predicted). **Right side**: Differences in samples between events based measured and predicted vertical GRF. One sample equal ~7 ms. MAE, mean absolute error; FC, Foot contact; FO, Foot off.

Estimation of gait events such as foot contact and foot off from vertical GRF waveforms are considered the gold standard in movement analysis. An example of the detection algorithm during walking and running can be found in Figure 2. One sample difference between the events based on the measured and predicted vertical GRF waveforms equaled ~7 ms due to the relatively low sampling frequency, common to wearable accelerometers.

### Statistical evaluation

The normalized root mean square error ϵ and the coefficient of determination *R*^2^ served for quality assessment of the predicted vertical ground reaction forces. We defined them as follows:


$$\begin{array}{l} \epsilon =\frac{\langle ||z-y|{|}_{2}\rangle }{\text{range}\left(z\right)}\;and\;R^{2}=1-\frac{\langle ||z-y|{|}_{2}^{2}\rangle }{\langle ||z-\langle z\rangle |{|}_{2}^{2}\rangle } \end{array}$$

Prediction of stepping moments were validated using the mean absolute error defined as


$$\begin{array}{l} \frac{1}{t}\sum_{i=1}^{t}\left|E_{\text{target},i}-E_{\text{prediction},i}\right| \end{array}$$

where, *E*_target,*i*_ and *E*_prediction,*i*_ refer to target and prediction events *i* = 1, …, *t*, respectively.

We evaluated the training via cross-validation with 50% of the data segmented and subsequently used for training, 25% continuous data for validation, and the remaining 25% continuous data for testing. We performed 100 repetitions with a random draw each time. A continuation rule was used, such that if the *R*^2^ of the validation data were all positive, the testing could be employed, and the training was satisfactory. A maximum of 100 repetitions were allocated for validation and in cases where the validation criteria was not satisfied, the training was stopped. In all cases, the number of strides used from each trial during training was reduced to 25 to ensure a balanced design.

**Figure 3:**
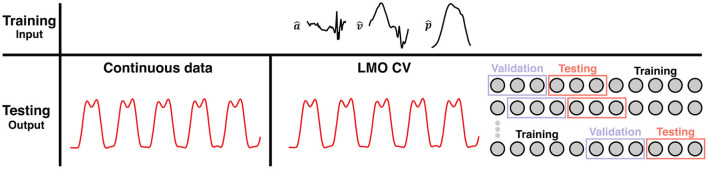
Schematic of the different testing scenarios used for validating the robustness of the reservoir computer. A random trace of a walking trial is shown here. The input data ($\hat{a},\hat{v},\hat{p}$) were first segmented into strides (we considered 36 samples in time on either side of the foot off as transients when correcting for any learning errors in the beginning or end of the data). The first scenario, the training is performed on segmented data and the testing on continuous data. The continuous data (in red) represents the output of the reservoir, the vertical GRF waveforms. Secondly, the training was performed on segmented data, testing was done on continuous, but a leave-M-out cross-validation (LMO CV) was employed (split: 50/25/25% for training, validation, and testing, respectively). The vertical GRF in red represents the output of the trained reservoir during testing. LMO CV, Leave-M-out cross-validation.

Two scenarios served to assess the robustness of the reservoir computer as sketched in Figure 3.

#### Training on segmented data—Validating and testing on continuous data

The applicability of our machine learning approach on continuous data were verified by training the reservoir computer on single strides and subsequent testing on continuous data from each trial (see Figure 3). First, we extracted a random 50% of continuous data from each trial before segmenting the remaining data into strides. The continuous data was split in two so 25% of the data were used for validation and testing, respectively. The segmented data were pooled across trials and conditions before being used for training. Training was validated by verifying the mean *R*^2^ over the validation set to be positive (see above for definition). Whenever validation did not pass with success, training was repeated using the same subset but other randomly chosen initial conditions (here in all cases validation was passed on first attempt). The entire process was repeated 100 times to allow for statistical evaluation as mentioned earlier.

Additionally, we estimated the minimum amount of data needed to secure a good reconstruction quality (*R*^2^ > 0.95), so the training data were reduced. We repeated the training 100 times from 4% of the total dataset to 50% of the total dataset. The validation and test sets remained 25% each for all runs (here the smaller training set sizes required re-learning but eventually validation was passed with success).

#### Leave-M-out cross-validation

To test the machine learner's ability to work as a classifier across participants, it was first trained and validated on a subgroup of participants and then tested on others that was unknown to the machine learner. The cross-validation split was performed based on trials. *M* trials were held out and the remaining 42-M trials were split 75/25% of the total dataset for training and validation. A total of 42 repetitions were performed. In the main text we report the result for *M* = 1 while the range *M* = 1, 2…, 6 is depicted in Supplementary Figure S2.

Unless specified otherwise, means and standard deviations are provided and were calculated as either the grand averages or the standard deviations across the 100 repetitions.

## Results

A total of 3,020 strides were included from 42 trials [1,249 walking strides (21 trials) and 1,771 running strides (21 trials)]. Here, we would like to note that we only show results of the right-side analysis, as the left-side results were very similar. Given this similarity one may pool data across sides to increase the sample size but, as will become clear, this was not needed.

**Figure 4:**
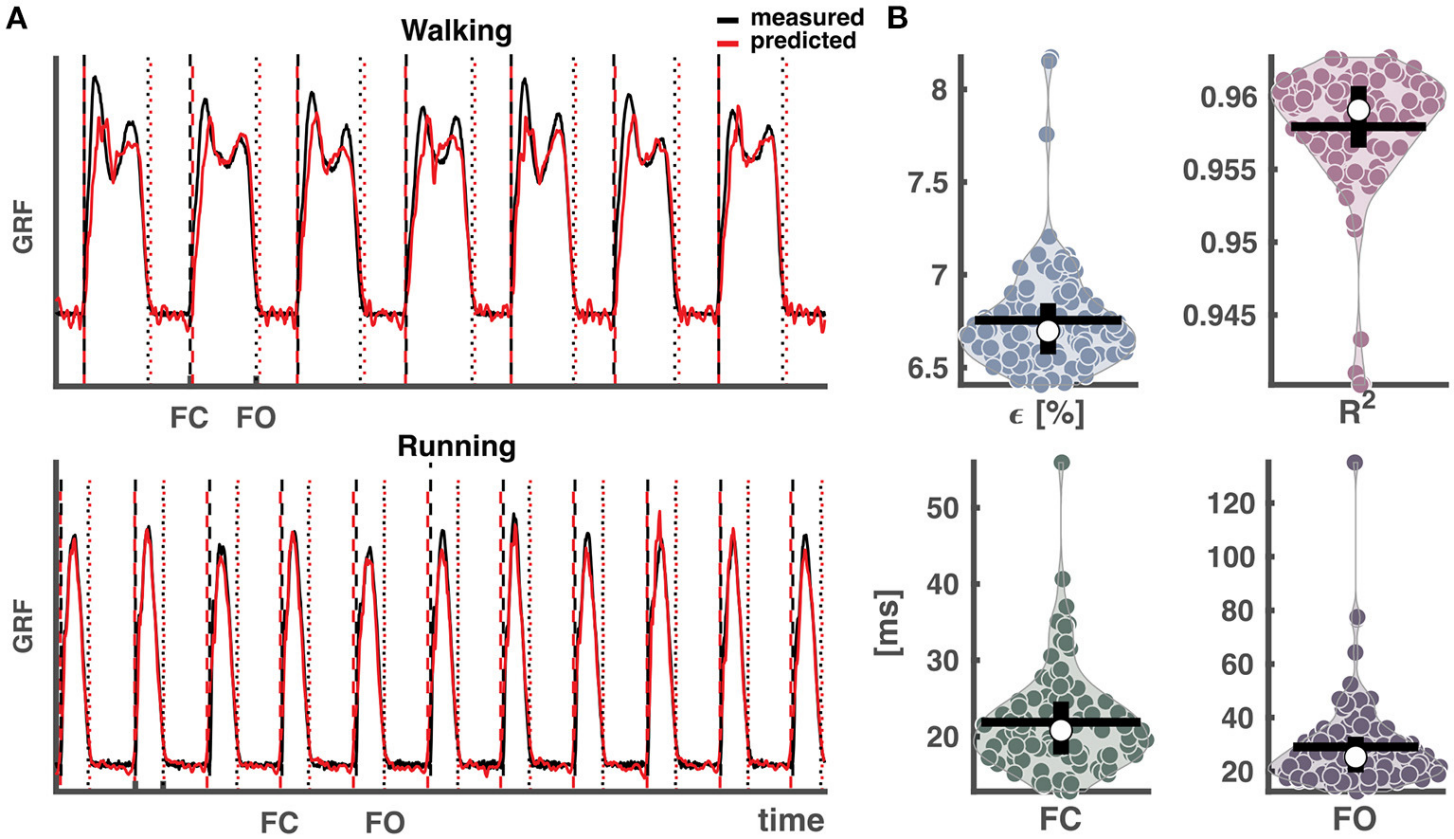
Output of reservoir computer trained on segmented data and validated and tested on continuous data pooled over conditions. **(A)** Vertical ground reaction force (GRF) waveforms of four randomly selected strides from each condition from a random trial and a random selected training run out of the 100, with the measured vertical GRF waveforms in black and predicted in red. The vertical dashed lines represent the foot contact events (black: measured, red: predicted), the vertical dotted lines the foot off events (black: measured, red: predicted). **(B)** Normalized root-mean squared error (**ϵ**), coefficient of determination (**R**^**2**^), mean absolute error of foot contact and foot off. The white dots in the violin-plots illustrate the medians. Black horizontal lines represent the mean and vertical black lines the 1st and 3rd quartiles. Every dot represents one of the 100 training runs, and the width of the violins is determined by the frequency. GRF, ground reaction forces; FC, foot contact; FO, foot off.

The performance of 100 repetitions in predicting GRF waveforms exceeded 95% when combining walking and running data. The coefficients of determination *R*^2^ were 0.96 ± 0.00 and the normalized root-mean squared errors ϵ were 6.8 ± 0.3% (mean ± SD) (cf. Figure 4). On average, the subsequently extracted foot contact and foot off events deviated from those based on the measured vertical GRF waveforms by 3 and 4 samples. This corresponds to mean absolute errors of 21.9 ± 6.5 ms and 29.1 ± 16.0 ms for foot contact and foot off events, respectively. Here we would like to add that the likewise convincing results when training the network on only walking or on only running are provided as Supplementary Figure S1.

**Figure 5:**
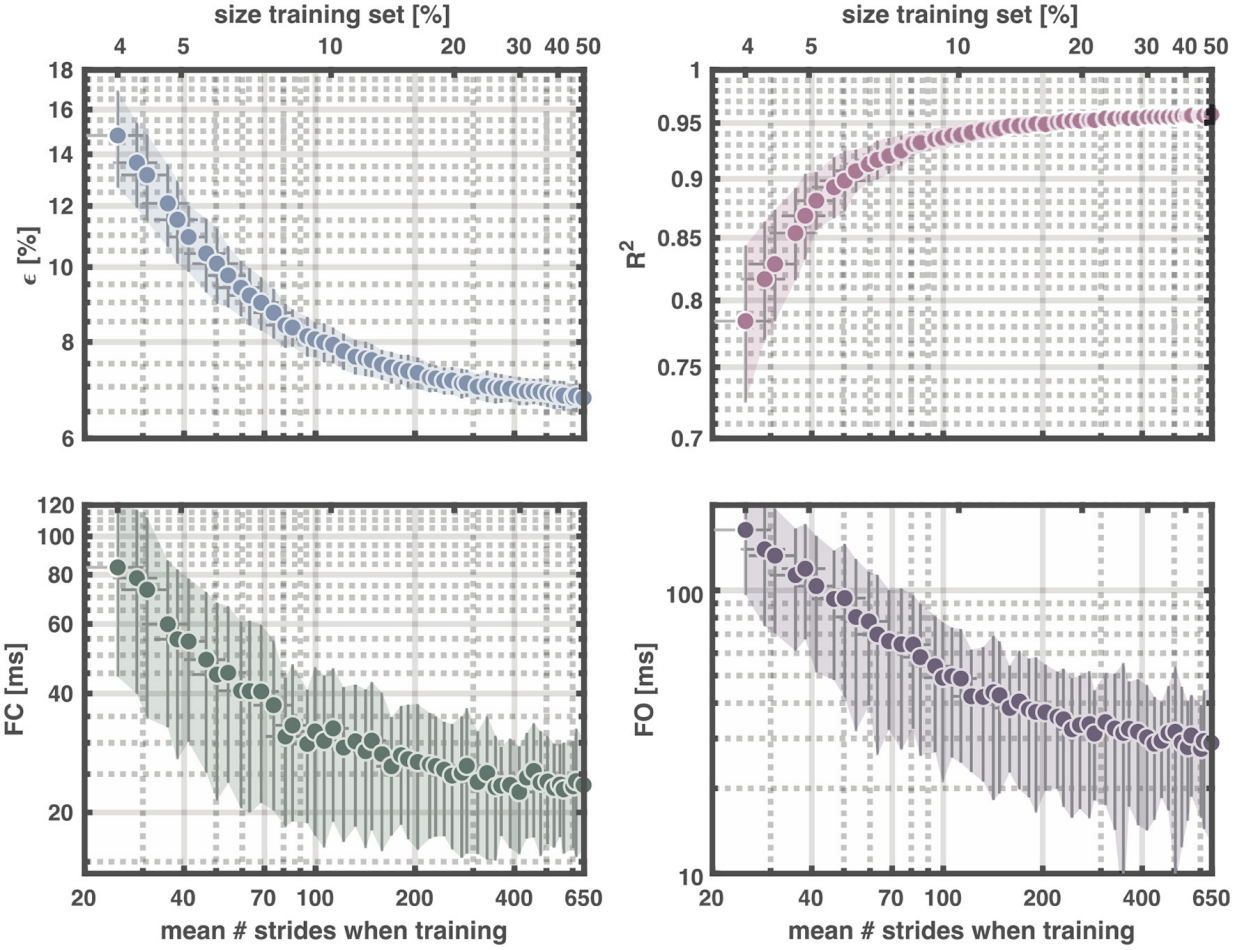
Output of reservoir computer with training data ranging from 4-50% of the total dataset and validating and testing with 25%, respectively. **Upper left panel**: Normalized root-mean squared error (**ϵ**), **upper right panel**: Coefficient of determination (**R**^**2**^), **lower left panel**: Mean absolute error of foot contact, and **lower right panel**: Mean absolute error of foot off. Upper x-axes show the percentage of the total dataset used for training and the lower x-axes the corresponding mean number of strides. Each dot represents the mean value for 100 repetitions. The vertical error-bars and colored areas represent the standard deviation of the corresponding measure. The horizontal error-bars represent the standard deviation in the number of strides across the 100 repetitions. FC, foot contact; FO, foot off.

To estimate the smallest number of strides needed for a mean reconstruction accuracy above 95%, we changed the size of the training set from 4 to 50% for the total dataset size (again with maximum 25 strides per trial for the training) (cf. Figure 5). The size of the validation and test sets were kept fixed at 25% each to guarantee identical accuracy demands. An average of ~222 strides, ~17% of the total dataset sufficed to reach *R*^2^ = 0.95 ± 0.01 with ϵ = 7.2 ± 0.3% and a mean absolute error of the foot contact (foot off) of 26.4 ± 9.3 ms (35.8 ± 15.8 ms).

Finally, to test whether our approach allows for predicting vertical GRFs in trials that in their entirety were not part of the learning set, we performed a leave-M-out cross-validation. Training was realized in the held-in trials using a 75–25% split of learning and validation (in the training set we used a maximum number of 25 strides per trial). For *M* = 1, the mean *R*^2^ reached 0.91 ± 0.12, with an error ϵ of 9.1 ± 3.6%. One trial was a clear outlier in the leave-one-out analysis, and when re-calculating the means without this trial, the mean *R*^2^ and the error ϵ were 0.93 ± 0.04 and 8.6 ± 2.3%, respectively. The corresponding mean absolute errors for foot contact and foot off were 63.7 ± 167.1 ms and 140.9 ± 224.3 ms for all included trials and 49.0 ± 138.9 ms and 129.8 ± 215.1 ms when not taking the outlier trial into account. For an overview of the results of *M* = 1 to *M*= 6, we refer to Supplementary Figure S2.

## Discussion

Reservoir computers are a promising tool to predict vertical GRF waveforms based on accelerometer data measured at the shank during walking and running. Accurately predicted GRF waveforms facilitate the detection of gait phases and events. We showed that this “simple” machine learning approach has excellent prediction accuracy of continuous vertical GRF waveforms independent of the type of locomotion. Put differently, reservoir computers can be used for predicting vertical GRF waveforms for gait of unknown type with excellent performance. This has great potential for uses outside the lab and for collecting large amounts of data. Without a doubt the growing amount of data available for biomechanical analysis in running will greatly drive the field forwards (76). Machine learning combined with wearable sensors may be the solution to increase the amount of data recorded.

Using machine learning for activity recognition and gait phase recognition based on gait features extracted from biomechanical data, which may be measured with wearable sensors (77, 78) has become increasingly popular. The most common techniques to classify gait events or predict GRF waveforms using machine learning are hidden Markov models (26, 30, 33, 34, 37, 43, 79–82), neural networks such as deep neural networks (more than 1 hidden layer) (25, 35, 36, 44, 47, 52); feed-forward neural networks (48, 50, 53, 56–58, 83, 84); long short-term models (24, 28, 38, 54, 83, 84); convolutional neural networks (29, 55); support vector machines (42, 44, 85); (multilayer) perceptron models (28, 42, 49, 51, 86), as well as random forest classifiers (36, 42, 44), K-nearest neighbors (42, 54, 87), and other types of machine learning using, e.g., Bayesian models (31, 32, 82, 85), Gaussian mixture model (41), and principal component analysis (39, 40, 51, 82). Reservoir computers have the great advantage of low computational costs while still showing excellent performance. They merely require a handful of time series for training and avoid any a priori feature extraction. Reservoir computers even seem to be promising as a tool to successfully reproduce locomotor patterns observed during walking and running (88).

There have been several studies conducted on predicting GRFs based on wearables, the vast majority for the stance phase only. Utilizing these machine learning techniques properly requires foot contact and foot off to be known. This, however, can only be accomplished by either co-registering footswitches or ground reaction forces, or by implementing a rule-based detection of the gait events. This is exactly what we sought to circumvent. Rule-based detections based on accelerometers during running, searching for a specific peak or valley in some area of interest, are not as precise as an event detection based on vertical ground reaction forces.

We did not restrict the prediction to only the stance phase—we included the entire gait cycle and showed that this worked well on continuous data. We also did not time-normalize the gait cycles and did not impose any other constraints into the timing of the signals. As such, our predictions are robust against variations in speed and stride durations/lengths as well as the type of locomotion. For training the reservoir computer, data were segmented based on the ground truth events, though when these are unknown, the data could be segmented in any way, or they may not be segmented at all (cf. Figure 4). We are convinced that our approach is suitable for lab as well as outdoor use. One very recent study (89) predicted continuous vertical GRFs from trunk accelerations using a long short-term model network with good accuracy during sloped running. The pre-processing involved several filtering and feature extraction steps. By contrast, here we succeeded to reduce the number of pre-processing steps and applied only very weak filtering, i.e., the inputs are by and large the time series (derived from) vertical acceleration.

We validated the use of accelerometer data to estimate the vertical GRF waveform. We used accelerometer data collected by a sensor that could, in fact, also be used for electromyography data collection. The sensors were not aligned in the same way for all participants, which Tan et al. (90) found can negatively affect the precision of the detection of GRFs using machine learning. However, variability in orientation and position is likely to occur if participants mount their own devices or in large-scale studies. By correcting the orientation of the accelerometers using principal component analysis we circumvent these potential problems and underline the robustness of our method and its applicability in many settings.

Even a small number of strides sufficed to achieve a high reconstruction accuracy. While strides from all participants were pooled for this analysis, it is unlikely that the strides randomly drawn into the training sets were representative for all trials/conditions and that this was also the case for the test sets. We evaluate this via leave-M-out cross-validation, as the test-set should be unknown to the machine (70). Admittedly, the reconstruction accuracy was not as high, but we consider it still satisfactory. The resulting event detection of the leave-M-out cross-validation, did not perform satisfactorily because of an introduction of jitter into the swing phase of the GRF which led many events to be detected too early or too late. We trust that a revision of the event detection algorithm can result in an improved foot strike and foot off estimation compared to the gold standard.

We would like to note that we did not optimize the machine learner to perform the absolute best it can do. Our primary aim was to show that even in its “simplest” form, a reservoir computer with ridge-regression-based output weights can perform well. Apparently, this (off-line) approach has its limits as, during learning, one must store all network states which can put pressure on computer memory. The alternative online learning may be realized via recursive least squares regression (91), that has recently be adopted by Sussillo and Abbott (92). Along these lines one may add online feedback and change the reservoir's connectivity for the network's dynamics to reach the chaotic regime (currently we used a spectral radius of 0.5 but values larger than 1 may accelerate online learning) (92, 93). For our proof-of-concept, however, fine-tuning the reservoir might be considered overfitting, which let us decide not to progress along this direction. The most optimal settings will probably depend on the data set under study.

Our accelerometers had a relatively low sampling rate (2,000/14 Hz ≈ 143 Hz), which prevents better estimation than 7 ms (i.e., one frame equals 7 ms). An accelerometer with higher sampling frequency will arguably lead to higher accuracy of the predicted events compared to the ground truth. A sampling frequency of 60–200 Hz is not uncommon when recording kinematics (94–97) and the accuracy is not worse than the accuracy one could obtain using kinematic data. A frequently employed detection algorithm for kinematic gait event detection is the coordinate-based detection algorithm where the distance between the sacrum and foot is used to predict foot contact and foot off events [(96), currently cited >800 times]. A review on this and other detection algorithms (97) during running revealed that the coordinate-based detection algorithm has an absolute error of 29 ms for foot contact and 98 ms for foot off (sampling frequency: 200 Hz) whereas the best performing algorithms has an accuracy of 24 ms for foot contact {the foot vertical position [(98), >600 citations]} and 6 ms for foot off {the peak knee extension algorithm [(99), >600 citations)]}. For comparison, the best estimation possible with a sampling frequency of 200 Hz is 5 ms, i.e., current algorithms have an accuracy between one and ~20 samples. Our approach is comparable to or exceeding this accuracy. Being cheap and easy to collect, being usable outside the lab and for long time-periods are, hence, not the only advantages of accelerometers—they also come with formidable accuracy in step detection when properly combined with reservoir computers.

Our machine learning approach performs well for a dataset comprised of both walking and running data despite a relatively small number of participants and a relatively small number of strides. To investigate its ability for each condition separately, we refer to Supplementary Figure S1, where we show that training and testing on only one type of locomotion improves the already excellent reconstruction accuracy. The outputs of the reservoir computer can easily be modified to provide other outputs such as other components of the GRF or the center-of-pressure. By including all three components of the GRF, energetics of the center-of-mass can be estimated during overground/track running which in turn can provide even more information about the locomotion type. Of course, the energetics will be in arbitrary units given our GRF prediction rely on normalize (z-scored) values. To expand the prediction from z-scored GRF to GRF containing information about the body weight of the participant, a more diverse group of participants are needed for training data. However, despite this shortfall, we believe that this is feasible. All data for this study were recorded on the treadmill. The next step will be to apply reservoir-based prediction to accelerometer data (or gyroscope data) obtained at other parts of the body, e.g., hip mounted (e.g., activity trackers), arm mounted (e.g., sport watches, smartphones) or head mounted (e.g., augmented/virtual reality glasses) to broaden applicability in daily living contexts as well as in clinical populations as machine learning algorithms might perform worse on clinical gait (100). This certainly calls for expanding the current dataset with overground/outdoor locomotion. We expect our findings to be transferable to overground settings. A large meta-analysis suggests that neither vertical ground reaction forces, nor peak tibial accelerations are significantly different between treadmill and overground running (101). However, this might not be true when accelerations, decelerations, turns, etc. are considered.

As a final note we would like to recall that our reservoir computer did not include a feedback loop. Adding feedback may allow for not only predicting the GRF accompanying tibial accelerations but eventually also the GRF in forthcoming strides. We trust that future studies will pursue this generalization as it is beyond the scope of our proof-of-concept study. Given our prediction results, however, we can stress that all the information needed for predicting (vertical) ground reaction forces seems to be present in the (principal component of) accelerometer signals. The use of the latter, hence, provides more opportunities than commonly thought.

Our data and code are made freely available and are ready to use on other datasets and can be extended for use in experiments and clinic.

## Conclusion

Reservoir computers are an excellent candidate to correctly predict vertical ground reaction force waveforms from accelerometer signals for a small number of participants and strides. The predicted time series can serve to estimate stepping moments with particularly high accuracy. The ease in training procedure, which requires only a (very) limited number of steps and without prior knowledge about the type of locomotion lets us advocate this machine learning approach to be further expanded to be applied on future applications in both research and clinic.

## Data availability statement

The dataset and code can be found via the following link: https://github.com/marlow17/PredictingGroundReactionForces.

## Ethics statement

The studies involving human participants were reviewed and approved by the Scientific and Ethical Review Board of the Faculty of Behavioural and Movement Sciences, Vrije Universiteit Amsterdam, Netherlands (File Number: VCWE-2022-008R1). The patients/participants provided their written informed consent to participate in this study.

## Author contributions

MB and ND set up the experiment. MB collected and pre-processed the data and prepared all figures. MB and AD wrote the code and the first draft of the manuscript. All authors commented and provided feedback on the final version of the manuscript.

## Funding

This project has received funding from the European Research Council (ERC) under the European Union's Horizon 2020 research and innovation program (Grant Agreement No. 715945 Learn2Walk) and from the Dutch Organization for Scientific Research (NWO) VIDI Grant (016.156.346 FirSTeps).

## Conflict of interest

The authors declare that the research was conducted in the absence of any commercial or financial relationships that could be construed as a potential conflict of interest.

## Publisher's note

All claims expressed in this article are solely those of the authors and do not necessarily represent those of their affiliated organizations, or those of the publisher, the editors and the reviewers. Any product that may be evaluated in this article, or claim that may be made by its manufacturer, is not guaranteed or endorsed by the publisher.

## Abbreviations

GRF: Ground reaction forces
ESN: Echo state network
PCA: principal component analysis.
